# Vitamin D in Follicular Fluid Correlates With the Euploid Status of Blastocysts in a Vitamin D Deficient Population

**DOI:** 10.3389/fendo.2020.609524

**Published:** 2021-01-25

**Authors:** Ana Arnanz, Neelke De Munck, Ibrahim El Khatib, Aşina Bayram, Andrea Abdala, Laura Melado, Barbara Lawrenz, Carol Coughlan, Alberto Pacheco, Juan A. Garcia-Velasco, Human M. Fatemi

**Affiliations:** ^1^ ART Fertility Clinic, Abu Dhabi/Dubai, United Arab Emirates; ^2^ Obstetrical Department, Women’s University Hospital Tuebingen, Tuebingen, Germany; ^3^ IVIRMA, Madrid, Spain; ^4^ Department of Reproductive Endocrinology and Infertility, Rey Juan Carlos University, Madrid, Spain

**Keywords:** PGT-A, 25(OH)D, bioavailable 25(OH)D, free 25(OH)D, % free 25(OH)D, follicular fluid

## Abstract

**Context:**

The widespread distribution of the Vitamin D (VitD) receptor in reproductive tissues suggests an important role for VitD in human reproduction. The assessment of patient´s VitD is based on the 25-hydroxyvitamin D (25(OH)D) metabolite measurement. However, most of the circulating 25(OH)D is bound to either VitD-binding protein (VDBP) (88%) or albumin (12%) and less than 1% circulates free.

**Objective:**

To determine a possible correlation between VitD levels in serum (S) and follicular fluid (FF) and blastocyst ploidy status in patients undergoing infertility treatment.

**Methods:**

A prospective observational study was performed including couples planned for preimplantation genetic testing for aneuploidies (PGT-A) from ART Fertility Clinics. Patients were classified according to their 25(OH)D-Serum levels: VitD deficient group <20 ng/ml and insufficient/replete ≥20 ng/ml defined as VitD non-deficient group.

**Results:**

Serum samples and 226 FF from individual follicles were collected for 25(OH)D, bioavailable 25(OH)D, free 25(OH)D, and % free 25(OH)D measurement. 25(OH)D-Serum in VitD deficient and non-deficient were 13.2±4.0 ng/ml vs 32.3±9.2 ng/ml; p<0.001. FF from 40 and 74 biopsied blastocysts was analysed of which 52.5 and 60.8% were euploid (p = 0.428), respectively. In VitD deficient patients, mean 25(OH)D-FF, bioavailable 25(OH)D-FF, and free 25(OH)D-FF were higher in euploid vs aneuploid blastocysts (18.3±6.3 ng/ml vs 13.9±4.8 ng/ml; p = 0.040; 1.5±0.5 ng/ml vs 1.1±0.4 ng/ml; p = 0.015; 0.005±0.002 ng/ml vs 0.003±0.001 ng/ml; p = 0.023, respectively), whilst no differences were found in VitD non-deficient patients (37.9±12.3 ng/ml vs 40.6±13.7 ng/ml; p = 0.380; 3.1±1.1 ng/ml vs 3.3±1.2 ng/ml; p = 0.323; 0.01±0.003 ng/ml vs 0.01±0.004 ng/ml; p = 0.319, respectively).

**Conclusion:**

VitD non-deficient patients have a significantly higher probability of obtaining a euploid blastocyst compared to VitD deficient patients (OR:33.36, p = 0.002).

## Introduction

**Figure 1:**
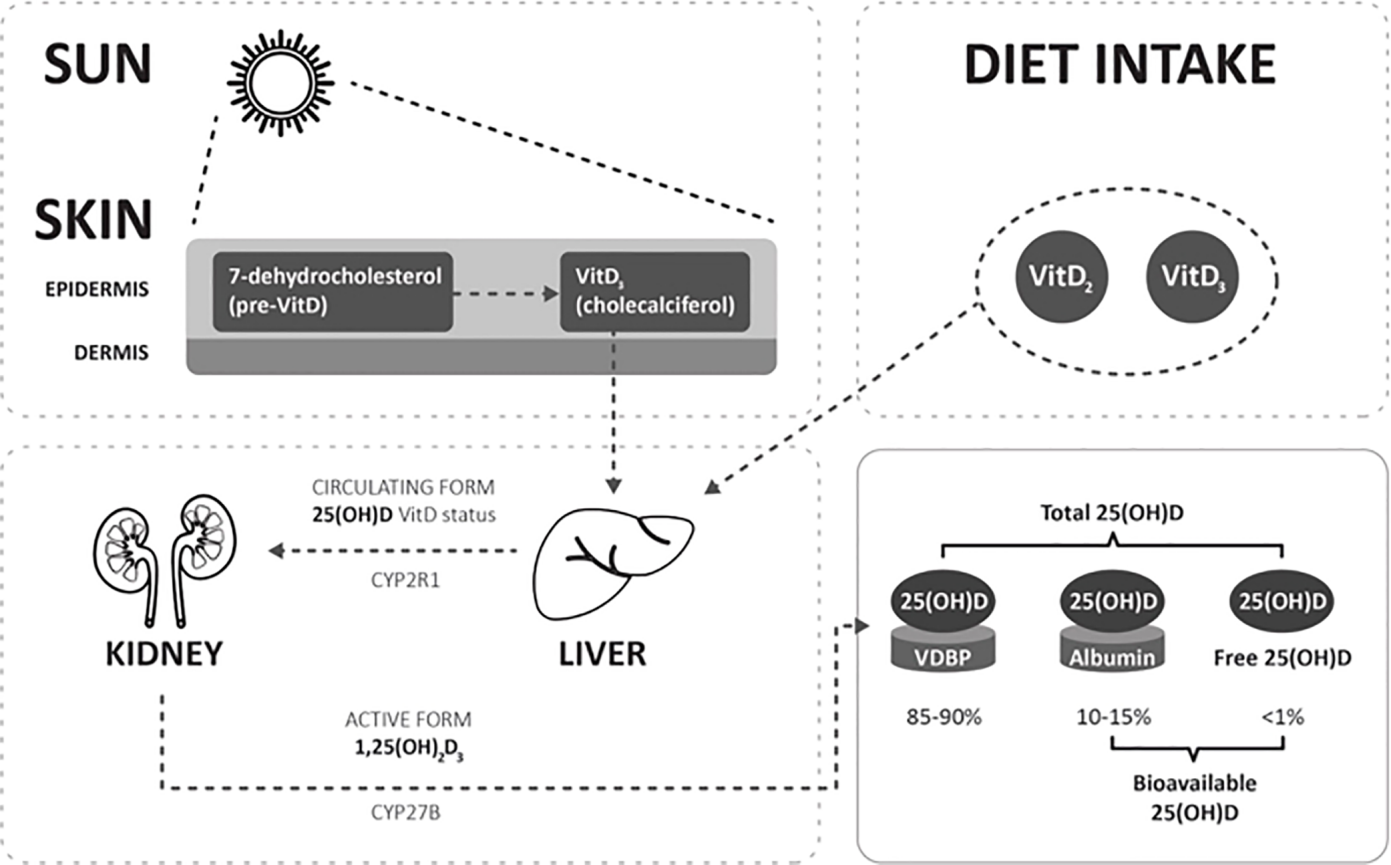
Simple schematic diagram depicting Vit D metabolism and the different Vit D metabolites: 25(OH)D, bioavailable 25(OH)D, and free 25(OH)D. VDBP, vitamin D binding protein; hepatic 25-hydroxylase, CYP2R1; renal 1α-hydroxylase, CYP27B1.

Vitamin D (VitD) is a steroid hormone known to control the musculoskeletal system by regulating the metabolism of calcium and phosphate. It is involved in many biological functions, mediated by the VitD receptor (VDR), a nuclear receptor that triggers two different cell signalling pathways (1, 2). More recently, VDRs have been identified in many reproductive tissues, including the ovaries (particularly granulosa cells), endometrium, placenta, testis, hypothalamus, and pituitary (3, 4) suggesting an important role for VitD in human reproduction and infertility (5, 6). The most important source VitD in humans is derived from exposure of the skin to sunlight (80–90%) while less than 10–20% is diet derived (7, 8) (**Figure 1**). When the skin is exposed to UVB light, 7- dehydrocholesterol (pre-VitD) transforms directly into VitD_3_ or cholecalciferol. After transport to the liver the first hydroxylation, by the hepatic 25-hydroxylase (CYP2R1) happens, that yields 25-hydroxyvitamin D (25(OH)D). Next, hydroxylation by the renal 1α-hydroxylase (CYP27B1) in the kidneys, produces the active 1,25-dihydroxyvitamin D_3_ form (1,25(OH)_2_D_3_). Most 25(OH)D is bound to the VitD binding protein (VDBP) or to serum albumin. The concentration not bound to VDBP is referred to as bioavailable 25(OH)D. Free 25(OH)D is the fraction not bound to VDBP or albumin, representing around 1% of total 25(OH)D, described as the biologically active hormone (9). The main circulating metabolite is 25(OH)D and serum 25(OH)D concentration is the best indicator of VitD nutritional status (10). According to the Endocrine Society, the VitD status of a patient is defined as deficient when 25(OH)D is below 20 ng/ml, insufficient when 21–29 ng/ml and replete if above 30 ng/ml (11).

The available evidence regarding the role of VitD in assisted reproduction remains conflicting. VitD appears to enhance the implantation process which has been attributed to the induction of immuno-tolerance (6, 12, 13). Studies of VitD in replete patients have shown a beneficial effect on implantation and clinical pregnancy rates (14, 15) while other studies were unable to document a benefit of VitD (16–21). In contrast, other studies have demonstrated that VitD insufficiency has an adverse effect on both clinical pregnancy and implantation rates (22–24). It is yet unclear as to whether VitD supplementation can counteract the reduced implantation rates demonstrated in VitD deficient patients (25).

The effect of VitD on folliculogenesis has also been studied by measuring 25(OH)D levels in serum and follicular fluid to determine if there is a correlation with IVF treatment outcomes. There is available evidence to suggest that the follicular biochemical environment may affect the development and quality of the oocyte (14, 15, 17, 21, 22, 26–28). Follicular fluid 25(OH)D reliably reflects serum levels (14) and studies suggest that women with replete serum 25(OH)D levels demonstrate higher fertilization rates (21), a relationship that is substantiated by the finding of lower fertilization rates in patients with VitD deficiency (15). However, despite the finding of lower fertilization rates in association with lower concentrations of follicular 25(OH)D, a study has shown that embryo quality is improved in patients with VitD deficiency, but embryo ploidy status was not assessed (29).

VitD deficiency is the most common vitamin deficiency worldwide and can be found in all ethnicities and age groups. Despite the abundance of sunlight and the concealing dress code owing to sociocultural/religious habits, the highest prevalence of VitD deficiency in the world is found in the Middle Eastern Arab population (96.9%) (30–32). Moreover, this population is characterized by low anti-Müllerian hormone (AMH) levels (33, 34). As previously shown, VitD has been suggested to be a positive regulator of AMH production (35–37), though the absence of any relation has also been reported (38–40). In order to fully understand the role of VitD in a predominantly VitD deficient Middle Eastern population undergoing fertility treatment, the present prospective pilot study explored the hypothesis of a possible correlation between serum and individual follicular fluid VitD levels [total 25(OH)D, bioavailable, free 25(OH)D and % free 25(OH)D] and embryonic competence and aneuploidy.

## Materials and Methods

Approval for this study was obtained from the Ethics Committee of IVIRMA Middle East Fertility Clinic, Abu Dhabi, UAE (United Arab Emirates) (Research Ethics Committee REFA007) and was registered at the ClinicalTrials.gov website (www.clinicaltrials.gov, trial number NCT03073720). Consents were obtained from every couple that participated in the study.

This prospective observational study was performed at IVIRMA Middle East, Abu Dhabi, UAE, between July 2017 and March 2019. Only Middle Eastern patients, undergoing intracytoplasmic sperm injection (ICSI) due to primary or secondary infertility and preimplantation genetic testing for aneuploidies (PGT-A) by next generation sequencing (NGS), were included in the study. Patients aged between 18 to 43 years old with a BMI of 19–33 kg/m^2^ and at least 6 follicles ≥14 mm at the time of trigger for final oocyte maturation were eligible for study inclusion. Exclusion criteria included severe male factor (WHO) and the use of cryopreserved sperm for ICSI. Further exclusion criteria included patients diagnosed with polycystic ovary syndrome in accordance with the Rotterdam criteria (41), a history of stage 3 or 4 endometriosis (as per the American Fertility Society) or a history of recurrent miscarriage (3 or more). Antimüllerian hormone (AMH), age and body mass index (BMI) values for all female partners were recorded. Patient’s VitD status was determined on the day of trigger for final oocyte maturation, by measuring levels of total 25(OH)D-Serum. Patients were classified into two groups according to their serum (S) 25(OH)D levels; VitD deficient <20 ng/ml (n = 13) and insufficient/replete ≥20 ng/ml (n = 24) defined as VitD non-deficient group.

**Figure 2:**
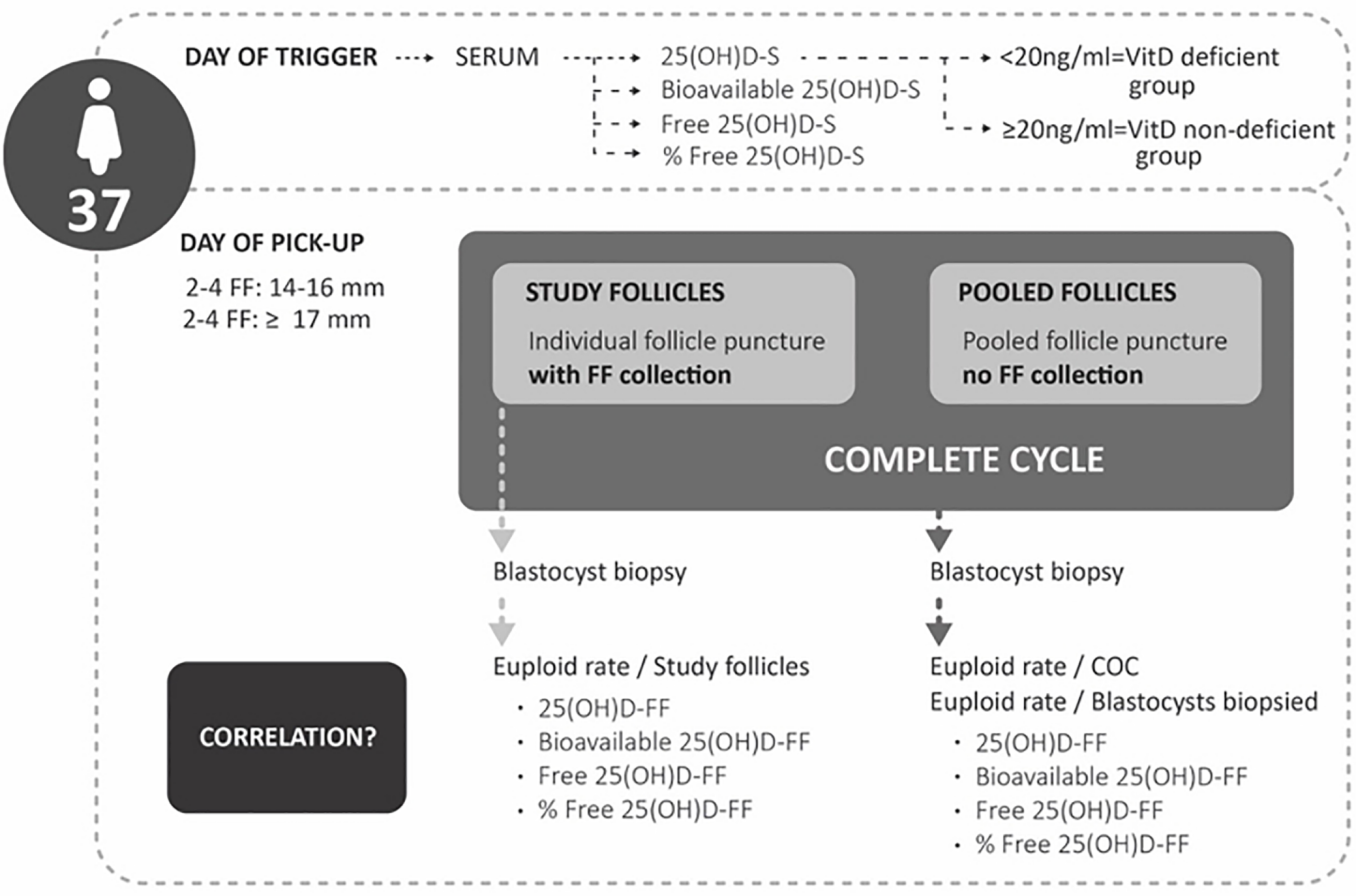
Study design.

The primary objective of the study was to determine if a correlation existed between VitD levels in individual follicular fluid (FF) and the ploidy status of the resulting blastocysts arising from the study follicles. In addition, the euploid rate defined as the number of euploid blastocysts per total number of blastocysts biopsied (from the study follicles) was determined. A further objective of this study was to ascertain whether serum VitD metabolites could predict euploid rate/cumulus oocyte complex (COC) retrieved, defined as the number of euploid blastocysts per COC retrieved (from the complete cycle) and euploid rate/blastocysts biopsied defined as number of euploid blastocysts/total number of blastocysts biopsied (from the complete cycle) (**Figure 2**). Moreover, the correlation between age and AMH in VitD deficient and VitD non-deficient patients was also assessed.

During oocyte retrieval (OR), at least two to four follicles between 14–16 mm and two to four follicles ≥17 mm were aspirated, and the FF aspirated from each follicle was matched to its retrieved oocyte. After being centrifuged at 800 g for 15 min, the supernatant was stored at -20°C. Once a minimum of four COCs were retrieved, the remaining follicles were aspirated and the obtained COCs were pooled together. Every mature oocyte (MII) retrieved from these individually punctured follicles was individualized, and the resulting embryos were monitored by time lapse imaging (Embryoscope ^TM^). All the analyses regarding serum are referred to as the complete cycle of the patient and the FF only to the study follicles.

### Hormone Assays

All serum and FF samples were stored at -20°C until analyses were performed. Serum AMH values were determined using a commercial fully automated assay by Elecsys® (Roche). Total 25(OH)D-Serum and 25(OH)D-FF values were measured in house, with Cobas e601 (Roche) using Electrochemiluminescence immunoassay (ECLIA) following the manufacturer’s recommendations. Limit of detection (LoD) and coefficient of variations were 3.0 ng/ml and 12.9% for serum, and 3.0 ng/ml and 3.8% for FF, respectively. An extra aliquot of frozen serum and FF samples was sent to IVIRMA Madrid where VitD binding protein and albumin were measured. A multiscan ELISA READER (Labsystems) measured VDBP using a commercial ELISA kit (R&D Systems) following the manufacturer´s recommendations. The limit of detection for VDBP was 0.15 ng/ml, the linearity limit was 250 ng/ml and the intra-inter assay coefficients of variations were 5.8 and 6.0%, respectively. Albumin was measured with a Cobas Mira Plus analyser (Roche). The LoD for albumin was 0.04 g/dl, the linearity limit was 6.0 g/dl, and the intra-/inter-assay coefficients of variation were 0.52 and 0.78%, respectively. Free and bioavailable 25(OH)D were determined using the method reported by Bikle et al. (42) applying a modified Vermeulen formula:

$$Calculated\;free\;25\;(OH)D=\frac{\text{Total}\;25\;(\text{OH})\text{D}}{1+[(6\text{x}105\text{x}[\text{Albumin}])+(7\text{x}108\text{x}[\text{VDBP}])]}$$

Bioavailable 25 (OH)D=(6x105x[Albumin]+1) x calculated free 25 (OH)D

[Albumin]=serum albumin in g/L÷66,430 g/mol

[VDBP]=serum VDPP in g/L÷58,000 g/mol

$$\%free\;25(OH)D=\frac{\text{Free}\;25(\text{OH})\text{D}}{\text{Total}\;25(\text{OH})\text{D}}$$

### Ovarian Stimulation Protocols

Ovarian stimulation was performed using standard protocols, either Gonadotropin Releasing Hormone (GnRH)- agonist or GnRH antagonist protocols with rFSH (recombinant Follicle Stimulating Hormone) or HP-HMG (Highly Purified Human Menopausal Gonadotropin) as stimulation medication. The dosage of the stimulation medication was decided based on individual ovarian reserve parameters (43). Trigger for final oocyte maturation was administered as soon as three follicles of at least 17 mm were present and oocyte retrieval was scheduled 36 h later. Oocyte retrieval was performed under ultrasound guidance as per routine clinical practice.

### Embryo Culture and Development

Denudation was performed 39 h post-trigger: the COCs retrieved from the study follicles were denuded individually and subsequently the MII oocytes were placed in separate culture drops until the time of the ICSI and the remaining pooled COCs (pooled follicles, not included in the study) were all denuded together (**Figure 2**). After ICSI, all inseminated oocytes were individually cultured in single step media (Global Total LP, CooperSurgical, Måløv, Denmark), overnight pre-equilibrated and maintained at the same incubation conditions 37°C, 5% O_2_, 6% CO_2_, and 89% N_2_. Fertilization was assessed 17–20 h post ICSI by the presence of two pronuclei. On day 3 of embryo development, culture medium was refreshed with 20 µl overnight pre-equilibrated Global total LP media (CooperSurgical, Måløv, Denmark).

Only expanded blastocysts (Gardner expansion grade 3–6) (44) with a clear and differentiated inner cells mass (ICM) and trophectoderm (TE) were subjected to trophectoderm biopsy on day 5, day 6, or day 7 of embryo development. Blastocyst scoring was based on the aspect, number and integrity of ICM and TE following the Spanish Asociación para el estudio de la Biología de la Reproducción (ASEBIR) consensus (45). Score A was given to a compacted ICM or TE made of many homogeneous cells that formed a tightly joined epithelium; score B to a loose aspect of the ICM or fewer TE cells that still formed a homogeneous epithelium; score C shows no signs of compaction in the ICM and very few TE cells and score D when ICM or TE cells showed signs of degeneration. Blastulation rate was defined as the number of cavitating blastocysts on day 5 per normally fertilized zygote.

Quinn’s Advantage Medium with HEPES (SAGE, CooperSurgical, Målov, Denmark) supplemented with HSA, (Vitrolife, Göteborg, Sweden) was used for the biopsy procedure. Three to five laser pulses on the zona pellucida (2.2 ms) along with mechanical “flicking” method were used to cut the trophectoderm cells inside the aspiration pipette; trophectoderm biopsies were washed and placed in 0.2 ml PCR tubes containing 2.5 μl PBS.

### Ploidy Status of Blastocysts by NGS

A whole genome amplification (WGA) protocol was performed on all individual samples (PicoPlex technology by Rubicon Genomics, Inc; Ann Arbor, Michigan, USA). After WGA, library preparation consisted of the incorporation of individual barcodes for the amplified DNA of each blastocyst. After isothermal amplification and enrichment, sequencing was performed on a 316 or 318 chip using the Personal Genome Machine sequencing (Life-Thermofisher, USA). Ion Reporter software, for sequencing analysis and data interpretation, was employed. The herein used NGS platform has been validated in previous studies (46, 47) and is commercially available.

All analyzed blastocysts were classified as euploid or aneuploid, no blastocysts with mosaicism or non-informative PGT-A results were reported.

### Statistical Analysis

Categorical data are presented as number and percentage. According to the distribution of continuous data, they are presented as mean ± SD (standard deviation) or median (range). The normality of distribution was checked using the Kolmogorov-Smirnoff test. Patient´s characteristics were compared using different tests according to the type of variable. The Student’s t test was used for normal continuous variables to find the difference between VitD deficient and VitD non-deficient patients. ANOVA test was used for non-normally distributed continuous variables and a mixed procedure with gamma distribution for non-normal variables such as rates. Logistic regression analysis was applied to test the predictability of VitD metabolites in FF and euploid status of the blastocysts. GLM procedure was used in FF and serum to assess the predictability of the VitD metabolites and the euploid rate/study follicles, euploid rate/COC and euploid rate/blastocyst biopsied. Spearman (Rh_0_) coefficient was used to test the strength of the correlation between age and AMH. A multivariate analysis was performed to find the factors associated to ploidy status. To consider inter and intra patient variability, the GENMOD procedure was used. For interpretation of the results, p value <0.05 was considered statistically significant. Non-estimated values, due to a small denominator, are represented as NE. All analyses were performed using SAS studio (Copyright © 2018 SAS Institute Inc., Cary, NC, USA).

## Results

### Cycle Characteristics

Patient´s characteristics are presented in **Table 1**. All 37 patients recruited for this prospective observational study were stratified according to 25(OH)D-Serum levels into VitD deficient (n = 13) and VitD non-deficient patients (n = 24), with mean 25(OH)D values of 13.2±4.0 ng/ml [median = 14.1 ng/ml (4.7–18.3)] vs 32.3±9.2 ng/ml [median = 29.4 ng/ml (21.72–56.08)], respectively. The age, BMI, AMH, follicle size, number of COCs, number of MII, blastulation rate and euploid rate for the study follicles as well as for the complete cycle did not vary among the groups.

**Table 1:** Patient´s characteristics.

	All patients	VitD deficient patients (n = 13)	VitD non-deficient patients (n = 24)	*p* value
Age (years old)	32.2 ± 5.8	31.3 ± 5.8	32.6 ± 5.8	0.477
BMI (kg/m^2^)	25.7 ± 3.7	26.1 ± 3.3	25.5 ± 3.9	0.645
AMH (ng/ml)	3.4 ± 1.7	3.3 ± 2.2	3.4 ± 1.4	0.878
Follicle size of study follicles at the time of OPU (mm)	16.9 ± 2.2	16.9 ± 2.3	17.0 ± 2.2	0.801
**Study follicles**				
Number of COCs	6.2 ± 0.9	6.1 ± 0.5	6.3 ± 1.1	0.443
Number of MII	5.5 ± 1.0	5.5 ± 0.9	5.5 ± 1.1	0.775
Blastulation rate	73.0 ± 30.5	65.0 ± 31.7	77.1 ± 29.7	0.195
Euploid rate	51.0 ± 30.5	49.2 ± 36	51.9 ± 34.6	0.828
**Complete cycle**				
Number of COCs	15.9 ± 6.0	17.0 ± 6.8	15.3 ± 5.7	0.431
Number of MII	13.0 ± 5.5	13.6 ± 6.7	12.7 ± 4.9	0.657
Blastulation rate	69.4 ± 29.5	63.0 ± 33.0	73.0 ± 28.0	0.290
Euploid rate	13.2 ± 7.0	12.0 ± 7.3	14 ± 7.0	0.413
Euploid rate/blastocysts biopsied	51.2 ± 30.5	44.8 ± 27.7	54.7 ± 32.0	0.351

Results are expressed as mean ± SD. SD, Standard deviation. t test for categorical data. COCs, cumulus oocytes complex; MII, mature oocyte; OPU, oocyte pick-up.

Out of 226 individually punctured follicles, a total of 114 were finally included in the study as they resulted in a biopsied blastocyst. The remaining 112 follicles were withdrawn from the study for multiple reasons; 12% (27/226) immature oocytes were retrieved and thus not injected, 23% (45/199) fertilized abnormally or failed to fertilize, 26% (40/154) resulted in arrested embryo development prior to blastocyst stage or the resulting blastocysts were of insufficient quality to be biopsied.

### Serum and FF Metabolites Between VitD Deficient and VitD Non-Deficient Patients

**Figure 3:**
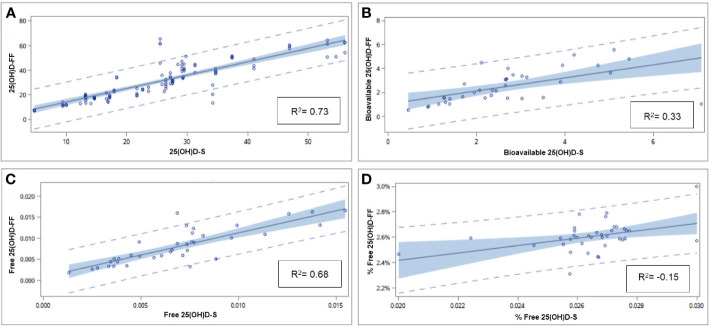
Linear regression between 25(OH)D **(A)**, bioavailable 25(OH)D **(B)**, free 25(OH)D **(C)**, and % free 25(OH)D **(D)** in S and FF. Linear regression model is presented with p values and coefficient of determination (R^2^). S, serum; F, follicular fluid.

Values of 25(OH)D, bioavailable 25(OH)D, free 25(OH)D, and % free 25(OH)D were measured in serum (n = 37) and FF (n = 114) and are presented in **Table 2**. Among VitD deficient and VitD non-deficient patients, significant differences were found between 25(OH)D-Serum, bioavailable 25(OH)D-Serum and free 25(OH)D-Serum (13.2±4.0 ng/ml vs 32.3±9.2 ng/ml; p < 0.001, 1.4±0.7 ng/ml vs 3.4±1.3 ng/ml; p < 0.001, 0.003±0.001 ng/ml vs 0.009±0.003; p < 0.001, respectively) as well as in FF (16.2±6.0 ng/ml vs 39.0±12.8 ng/ml; p <.001, 1.3±0.5 ng/ml vs 3.2±1.1 ng/ml; p <.001, 0.004 ng/ml ±0.0001 ng/ml vs 0.010±0.003 ng/ml; p <.001, respectively). However, % free 25(OH)D values did not vary in serum and FF between VitD deficient and VitD non-deficient patients (0.03±0.002 vs 0.03±0.002; p = 0.693 and 0.03±0.001 vs 0.02±0.001; p = 0.053, respectively). There was a significant linear association between the values of 25(OH)D, bioavailable 25(OH)D, free 25(OH), and % free 25(OH)D in serum and FF (r^2^ = 0.73, p <.0001; r^2^ = 0.33, p <.0001; r^2^ = 0.68, p <.0001 and r^2^ = 0.15, p = 0.009, respectively) (**Figure 3**).

**Table 2:** Association between Vitamin D metabolites in serum (S) and follicular fluid (FF) in VitD deficient patients and VitD non-deficient patients.

	All	VitD deficient patients	VitD non-deficient patients	*p* value
**Serum**				
25(OH) D-Serum (ng/ml)	25.6 ± 12.0	13.2 ± 4.0	32.3 ± 9.2	<0.001
bioavailable 25(OH)D-Serum (ng/ml)	2.7 ± 1.4	1.4 ± 0.7	3.4 ± 1.3	<0.001
Free 25(OH)D-Serum (ng/ml)	0.01 ± 0.003	0.003 ± 0.001	0.009 ± 0.003	<0.001
% free 25(OH)D-Serum (%)	0.03 ± 0.002	0.03 ± 0.002	0.03 ± 0.002	0.693
**Follicular fluid**				
25(OH) D-FF (ng/ml)	31.0 ± 15.4	16.2 ± 6.0	39.0 ± 12.8	<0.001
bioavailable 25(OH)D-FF (ng/ml)	2.5 ± 1.3	1.3 ± 0.5	3.2 ± 1.1	<0.001
Free 25(OH)D-FF (ng/ml)	0.01 ± 0.004	0.004 ± 0.0001	0.010 ± 0.003	<0.001
% free 25(OH)D-FF (%)	0.02 ± 0.001	0.03 ± 0.001	0.02 ± 0.001	0.053

Results are expressed as mean ± SD. SD, Standard deviation. t test for categorical data. L20 = 25(OH)D-S <20ng/ml and H20 = 25(OH)D-S ≥20ng/ml.

### Correlation Between Serum Metabolites and Euploid Status of the Complete Cycle

Serum VitD metabolites both in VitD deficient and VitD non-deficient patients, were not identified as predictors of euploid rate/COC (p > 0.05) (**Table 3**). However, once a blastocyst was biopsied in VitD deficient patients, 25(OH)D-Serum and bioavailable 25(OH)D-Serum were identified as predictors of euploid rate/blastocysts biopsied (p = 0.048 vs p = 0.0013, respectively). Only bioavailable 25(OH)D-Serum was a significant predictor of euploid rate/blastocysts biopsied in VitD non-deficient patients (p = 0.004).

**Table 3:** Predictability of serum Vitamin D metabolites (ng/ml) and euploid rate per blastocysts biopsied in VitD deficient and VitD non-deficient patients.

		Euploid rate/COC *p* value	Euploid rate/blastocysts biopsied *p* value
25(OH)D-Serum (ng/ml)	VitD deficient	0.453	0.048
	VitD non-deficient	0.656	0.132
Bioavailable 25(OH)D-Serum (ng/ml)	VitD deficient	0.771	0.013
	VitD non-deficient	0.842	0.004
Free 25(OH)D-Serum (ng/ml)	VitD deficient	0.437	0.097
	VitD non-deficient	0.661	0.198
% Free 25(OH)D-Serum	VitD deficient	0.885	0.308
	VitD non-deficient	0.989	0.709

Euploid rate/COC = number of euploid blastocysts per COC retrieved.

euploid rate/blastocysts biopsied = number of euploid blastocysts/total number of blastocysts biopsied.

COC, cumulus oocyte complex; S, serum; FF, follicular fluid.

VitD deficient = 25(OH)D-Serum <20ng/ml.

VitD non-deficient = 25(OH)D-Serum ≥20ng/ml.

### Correlation Between FF Metabolites and Euploid Status/Rate in Individual Follicles

**Figure 4:**
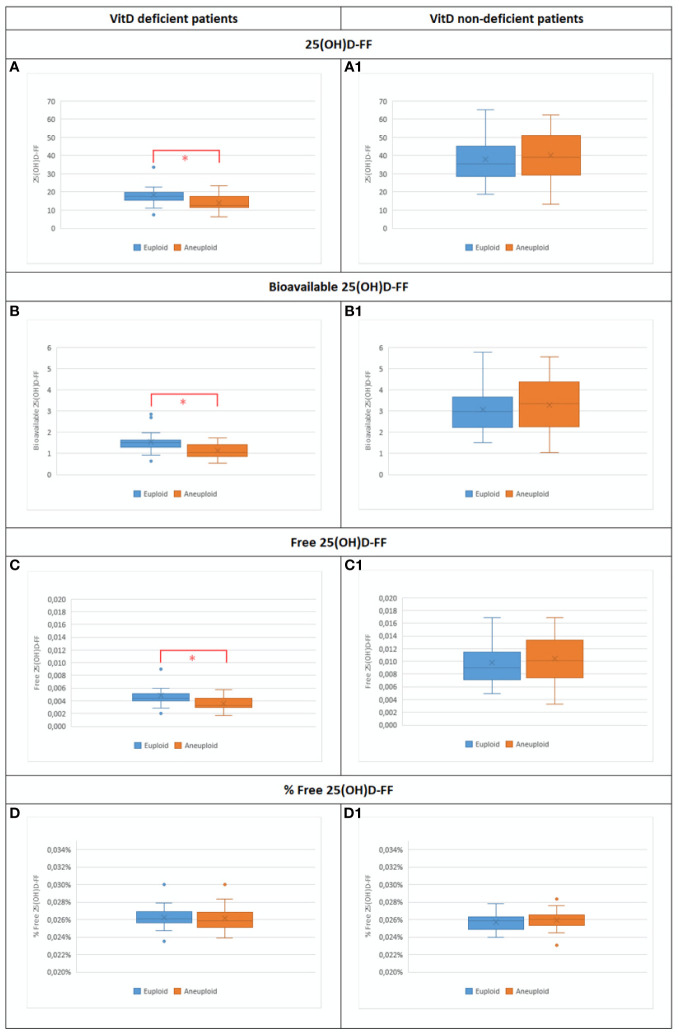
Bar plot representing the different Vit D metabolites in both groups of patients: Vit D deficient and Vit D non-deficient among euploid vs aneuploid blastocysts. **(A)** 25(OH)D-FF values in Vit D deficient euploid (n=21) vs Vit D deficient euploid (n=19) (18.3±6.3 vs 13.9±4.8;p=0.040*). (A1) 25(OH)D-FF values in Vit D non-deficient euploid (n=45) vs Vit D non-deficient aneuploid (n=29) (37.9±12.3 vs 40.6±13.7; p=0.380). **(B)** Bioavailable 25(OH)D-FF values in Vit D deficient euploid (n=21) vs Vit D deficient euploid (n=19) (1.5±0.5 vs 1.1±0.4; p=0.015*). (B1) Bioavailable 25(OH)D-FF values in Vit D non-deficient euploid (n=45) vs Vit D non-deficient aneuploid (n=29) (3.1±1.1 vs 3.3±1.2; p-0.323). **(C)** Free 25(OH)D-FF values in Vit D deficient euploid (n=21) vs Vit D deficient euploid (n=19) (0.005±0.002 vs 0.003±0.001; p=0.023*). (B1) Free 25(OH)D-FF values in Vit D non-deficient euploid (n=45) vs Vit D non-deficient aneuploid (n=29) (0.01±0.003 vs 0.01±0.004; p=0.319). **(D)** % Free 25(OH)D-FF values in Vit D deficient euploid (n=21) vs Vit D deficient euploid (n=19) (0.03±0.001 vs 0.03±0.001; p=0.783). (B1) % Free 25(OH)D-FF values in Vit D non-deficient euploid (n=45) vs Vit D non-deficient aneuploid (n=29) (0.02±0.001 vs 0.02±0.001;p=0.268).

A total of 114 blastocysts were biopsied: 40 blastocysts from VitD deficient patients and 74 blastocysts from VitD non-deficient group, yielding a euploid rate of 52.5% (21/40) and 60.8% (45/74), p = 0.428, respectively. A logistic procedure was used to measure the predictability of 25(OH)D-FF, bioavailable 25(OH)D-FF, free 25(OH)D-FF and % free 25(OH)D-FF in the study follicles and euploid status of the blastocysts between the groups. The results showed that, in VitD deficient patients, 25(OH)D-FF, bioavailable 25(OH)D-FF and free 25(OH)D-FF were the only predictors of euploid status [OR = 1.18 (1.01–1.38); p = 0.040, OR = 10.92 (1.58–75.56); p = 0.015 and OR = NE (NE-NE); p = 0.023 respectively], but not in VitD non-deficient patients (**Table 4**) (**Figure 4**). Similar findings were observed when analysing euploid rate/study follicles in VitD deficient patients; 25(OH)D-FF, bioavailable 25(OH)D-FF and free 25(OH)D-FF were also predictors of euploid rate (p = 0.008, p = 0.003 vs p = 0.006), respectively (**Table 4**).

**Table 4:** Predictability of follicular fluid Vitamin D metabolites (ng/ml) and euploid status/rate in individual follicles of VitD deficient patients and VitD non-deficient patients.

		Euploid status of the blastocysts					Euploid rate/study follicles
		Euploid	Aneuploid	OR	95% CI	*p* value	*p* value
25(OH)D-FF (ng/ml)	VitD deficient	18.3 ± 6.3	13.9 ± 4.8	1.18	1.01–1.38	0.040	0.008
	VitD non- deficient	37.9 ± 12.3	40.6 ± 13.7	0.98	0.94–1.02	0.380	0.329
Bioavailable 25(OH)D-FF (ng/ml)	VitD deficient	1.5 ± 0.5	1.1 ± 0.4	10.92	1.58–75.56	0.015	0.003
	VitD non- deficient	3.1 ± 1.1	3.3 ± 1.2	0.81	0.52–1.24	0.323	0.260
Free 25(OH)D-FF (ng/ml)	VitD deficient	0.005 ± 0.002	0.003 ± 0.001	NE	NE-NE	0.023	0.006
	VitD non- deficient	0.01 ± 0.003	0.01 ± 0.004	< 0.001	<0.001-NE	0.319	0.258
% free 25(OH)D-FF (%)	VitD deficient	0.03 ± 0.001	0.03 ± 0.001	NE	<0.001-NE	0.783	0.314
	VitD non- deficient	0.02 ± 0.001	0.02 ± 0.001	< 0.001	<0.001-NE	0.268	0.059

Euploid status of the blastocysts; Euploid vs Aneuploid. Results are expressed as mean ± SD. SD, Standard deviation; OR, Odds Ratio; 95% CI (confidence interval).

Euploid rate/study follicles = number of euploid blastocysts/total number of blastocysts biopsied (from the study follicles). VitD deficient patients = 25(OH)-Serum <20ng/ml. VitD non-deficient patients = 25(OH)D-Serum ≥20ng/ml.

NE, not estimated.

### Factors Affecting Euploid Rate in Study Follicles

A multiple regression model, taking into account intra and inter-patient variability in study follicles, revealed that after controlling for age, BMI and AMH, an increase of 1 unit in 25(OH)D-FF and bioavailable 25(OH)D-FF in VitD deficient patients, increased the odds of having a euploid blastocyst by 1.15 and 7.57 respectively (OR = 1.15 [1.02–1.30]; p = 0.017 and OR = 7.57 [2.21–25.96]; p = 0.001). In general, VitD non-deficient patients had higher probabilities of having a euploid embryo [OR = 33.36 (3.7–300.76); p = 0.002 and OR = 50.34 (6.04–364.96); p < 0.001]. Analyzing the interaction between VitD metabolites and the VitD status of the VitD non-deficient patients, with every unit increase in 25(OH)D-FF and bioavailable 25(OH)D-FF the odds of having a euploid blastocyst decreased [OR = 0.84 (0.75–0.95); p = 0.009 and OR = 0.10 (0.03–0.36); p < 0.001, respectively] (**Table 5**).

**Table 5:** Multiple regression model in VitD deficient and VitD non-deficient patients.

	25 (OH)D-FF			Bioavailable 25 (OH)D-FF			Free 25 (OH)D-FF		
VitD metabolite	OR	95% CI	p value	OR	95% CI	p value	OR	95% CI	p value
	1.15	1.02–1.30	0.017	7.57	2.21–25.96	0.001	0.0	0.0–0.0	0.009
BMI	1.01	0.92–1.12	0.705	1.01	0.92–1.12	0.797	0.98	0.89–1.08	0.695
Age	0.93	0.87–1.00	0.063	0.93	0.87–1.00	0.095	1.06	0.99–1.14	0.067
AMH	1.01	0.83–1.23	0.865	1.01	0.83–1.23	0.947	0.99	0.81–1.21	0.924
Status VitD	33.36	3.7–300.76	0.002	50.34	6.94–364.96	< 0.001	0.02	0.002–0.203	< 0.001
VitD metabolite-status VitD	0.84	0.75–0.95	0.009	0.10	0.03–0.36	< 0.001	NE	NE -NE	< 0.001

Reference group is VitD non-deficient, modelling for a euploid embryo. Results are expressed as OR, Odds Ratio; 95% CI, confidence interval and p values.

BMI, body mass index; AMH, anti-Müllerian hormone.

Status VitD: VitD status of the patient, VitD non-deficient compared to VitD deficient.

VitD metabolite-status VitD: Interaction between each VitD metabolite 25(OH)-D-FF, bioavailable 25(OH)-D-FF and free 25(OH)-D-FF and the status of the patient (VitD non-deficient as a reference).

**Figure 5:**
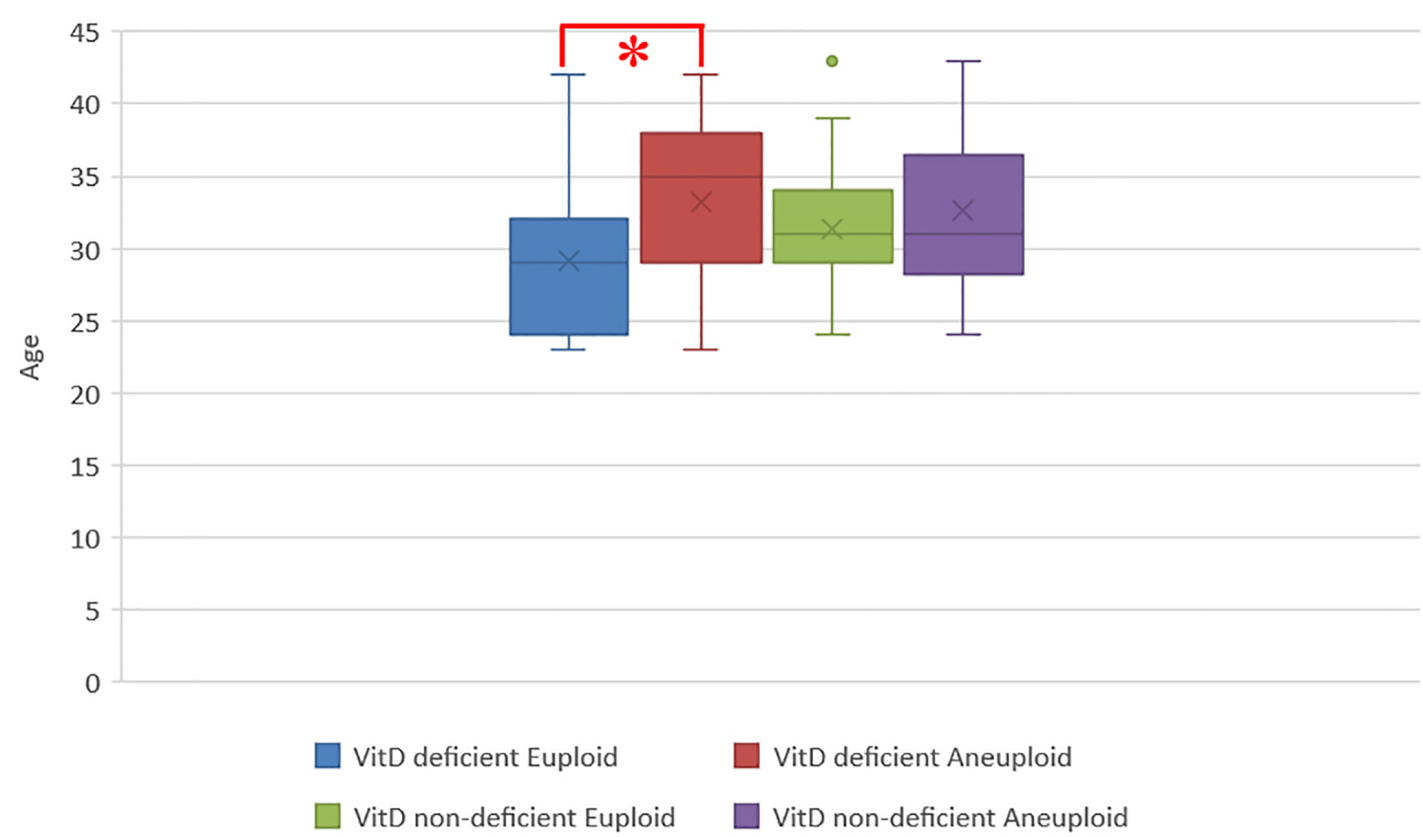
Bar plot showing the distribution of age among all biopsied blastocyst in H20 and L20 groups. Each bar represents age ± SD (years); Vit D deficient euploid (n=21) vs Vit D deficient aneuploid (n=19) (29.2±5.8 vs 33.3±5.5; p=0.03*), Vit D non-deficient euploid (n=45) vs Vit D non-deficient aneuploid (n=29) (31.4±4.3 vs 33.0±5.8; p=0.18).

Interestingly, when analyzing female age as a dependent variable of the blastocyst ploidy status between both groups, age was significantly lower in the VitD deficient study population with euploid blastocysts (29.2±5.8 vs 33.3±5.5, p = 0.03) but not in the VitD non-deficient group (31.4±4.3 vs 33.0±5.8, p = 0.18) (**Figure 5**).

**Figure 6:**
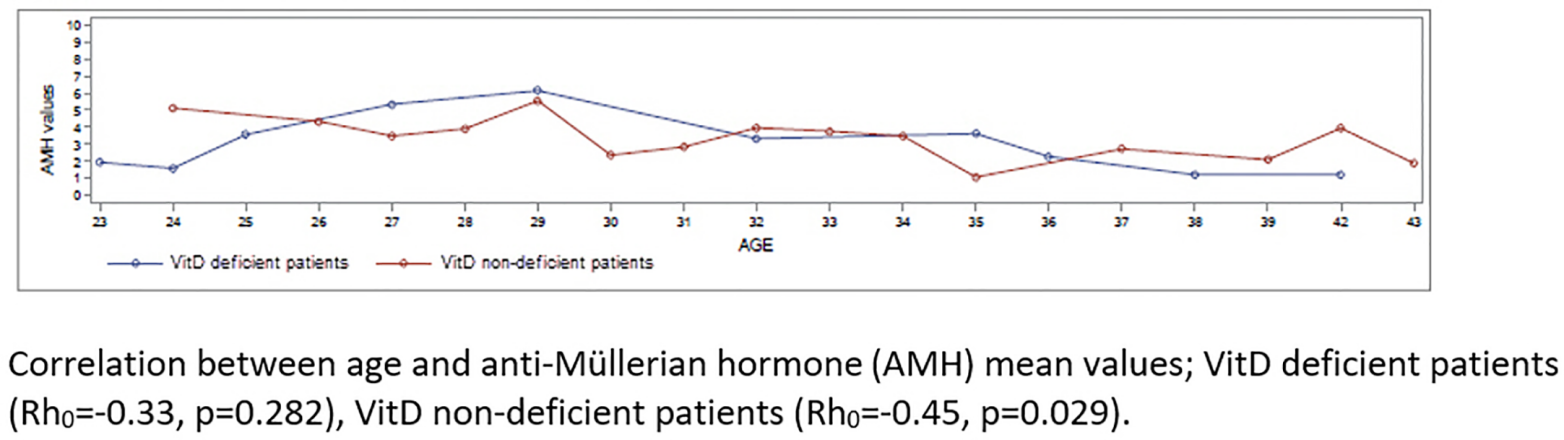
AMH and age distribution in Vit D deficient and Vit D non-deficient groups. Correlation between age and anti-Müllerian hormone (AMH) mean values; Vit D deficient patients (Rh0=-0.33, p=0.282), Vit D non-deficient patients (Rh0=-0.45, p=0.029).

In a Spearman test aiming to correlate age and AMH values, VitD deficient patients showed no correlation between age and AMH (Rh_0_ = -0.33, p = 0.282) whereas a significant negative correlation was observed for VitD non-deficient patients (Rh_0_ = -0.45, p = 0.029,) (**Figure 6**).

## Discussion

The current prospective observational study evaluated a possible correlation between 25(OH)D, bioavailable 25(OH)D, free 25(OH)D, and % free 25(OH)D values in serum and FF and the ploidy status of the resulting blastocysts. Based on the study findings it is interesting to note that being a VitD non-deficient patient increases the probability of having a euploid blastocyst (OR = 33.36), even more so than female age. Moreover, among VitD deficient patients, higher 25(OH)D-Serum/FF, bioavailable 25(OH)D-Serum/FF and free 25(OH)D-FF levels, in addition to younger female age, were positively correlated with euploid blastocysts.

The challenge among IVF experts is to understand how VitD plays a role in reproductive outcomes since there are many conflicting reports. Is there a potential effect on folliculogenesis and oogenesis or has it an effect on the endometrial receptivity affecting embryo implantation or on both? As the composition of FF provides the biochemical environment for the growth of the oocyte, it may affect the oocyte and embryo quality (26). However, studies that correlate total 25(OH)D, bioavailable 25(OH)D, free 25(OH)D, and % free 25(OH)D in individual FF with embryonic competence are currently lacking. Bioavailable 25(OH)D is a biomarker that is more robust than 25(OH)D which allows us to control for differences in VDBP concentrations among different races (48). Bioavailable 25(OH)D is the small fraction not bound to VDBP and it has been described that VDBP acts as a reservoir for the VitD metabolites, reducing the risk of VitD deficiency (49). The free hormone hypothesis states that protein-bound hormones are relatively inactive, while hormones not bound to binding proteins are able to cross the cell membrane and exert their biological activities (50, 51). Furthermore, although free 25(OH)D represents less than 0.1% of the total 25(OH)D, it might be more important for biological activity in most tissues (52). The specific action exerted by the increased amount of free active hormones on euploidy outcomes, needs to be further explored and should be interpreted carefully. In our study, the calculation of free 25(OH)D was based on a mathematical formula that takes into account total 25(OH)D values and the affinity constants from albumin and VDBP measurement, whose concentrations have been reported to be affected by a number of factors including medication intake, hormonal influences and smoking (53). Previous studies reported a significant correlation between directly measured free 25(OH)D (immunoassays) and total 25(OH)D in serum (52).

As previously shown by different authors, VitD levels in serum and FF were highly correlated suggesting that serum levels may act as surrogate marker for follicular fluid levels (14, 19). However, it is important to note that they did not differentiate between total 25(OH)D and its different forms; bioavailable 25(OH)D, free 25(OH)D, and % free 25(OH)D. Our results clearly demonstrated that not only levels of 25(OH)D-Serum are a reliable reflection of the levels measured in FF but also bioavailable 25(OH)D-Serum, free 25(OH)D-Serum and % free 25(OH)D-Serum. Regardless of the VitD status of the patient, levels of % free 25(OH)D in serum and FF remained similar. In serum of VitD deficient patients, 25(OH)D and bioavailable 25(OH)D were the key metabolites to predict ploidy of biopsied blastocysts. In other words, if the patient is VitD deficient and has a blastocyst biopsied, higher 25(OH)D-Serum and bioavailable 25(OH)D-Serum values will result in a higher chance to obtain a euploid blastocyst. Higher bioavailable 25(OH)D-Serum levels are also important in VitD non-deficient group to achieve a better euploid rate once a blastocyst is biopsied. The importance of increased VitD levels on ploidy outcome may explain the higher incidence of miscarriages in VitD deficient patients when transferring day 3 embryos not tested for aneuploidies (29).

In FF, the current results showed that in a VitD deficient population, levels of 25(OH)D-FF, bioavailable 25(OH)D-FF and free 25(OH)D-FF are not only predictors of blastocyst euploid status but also of the euploid rate in study follicles. Our study indicates that the follicular VitD environment impacts on embryonic ploidy outcome. Although intrafollicular VitD concentration has been suggested to be a predictor of embryo development (54), no differences were found in blastulation rate in the study follicles in VitD deficient vs VitD non-deficient study groups. It seems that VitD levels are metabolized differently in individual follicles, ultimately affecting the utilization of 25(OH)D-FF. Consequently, an adverse association between 25(OH)D-FF and oocyte competence cannot be drawn if FF is only retrieved from the leading follicle, as previously shown (29). In line with previous publications, in the VitD deficient group, female age was significantly higher in those whose blastocysts were found to be aneuploid, as compared to those with blastocysts of normal ploidy status, highlighting the adverse effect of advancing female age on blastocyst ploidy status particularly if the patient is VitD deficient.

An increase of 1 unit in 25(OH)D-FF, bioavailable 25(OH)D-FF or free 25(OH)D-FF in VitD deficient patients, increased the probability to have a euploid blastocyst. Similar results were shown by Ozkan and colleagues, who demonstrated that each gain of 1 ng/ml of VitD follicular fluid increased the probability of a clinical pregnancy by 6% (p = 0.030) (14). When comparing VitD non-deficient with VitD deficient patients, 25(OH)D-FF, bioavailable 25(OH)D-FF and free 25(OH)D-FF were the only parameters able to predict ploidy. Interestingly, analyzing the interaction between 25(OH)D-FF and VitD non-deficient as a reference group for the status of the patient, every unit increase in 25(OH)D-FF, bioavailable 25(OH)D-FF and free 25(OH)D-FF decreases the probability of having a euploid blastocyst. This finding is also in line with what was previously reported; insufficient/replete patients with increased levels of 25(OH)D-FF may have an adverse effect on embryo development, particularly when combined with a decrease in glucose levels (17). Locally elevated 25(OH)D concentrations may be the result of a lower VitD receptor (VDBP) affinity or of weakened 1α-hydroxylase activity or other mechanisms that affect VitD conversion to its active form in the individual follicle, having an adverse effect on oocyte quality.

Previous studies, with a lower proportion of the study population being VitD deficient (18.4 and 30.7%), observed no correlation between VitD status and AMH (38, 39). However, it has been suggested that VitD modifies AMH signaling and steroidogenesis in human cumulus-granulosa cells, indicating that both variables should be correlated (5). A negative linear correlation was observed between AMH levels in serum and FF and total VitD concentrations up to approximately 30 ng/ml; with a statistically significant relationship in FF (55). The study of Zhengfen and colleagues (2019) (34) revealed that low levels of VitD may contribute to decreased ovarian reserve. Surprisingly, the VitD deficient patients in our study showed a non-age related AMH distribution, while the AMH of VitD non-deficient patients showed the known linear decrease with age.

In the Middle East, despite the abundance of sunlight, the prevalence of VitD deficiency is high and most patients are receiving vitamin D2 (ergocalciferol) supplementation. Since they do not regularly monitor their vitamin intake, we were unable to assess individual supplementation or not, nor do we know the nutritional deficiency. Nevertheless, randomized controlled trials are needed to test whether VitD supplementation in a VitD deficient population can improve their fertility (24). Our study is strengthened by the homogeneity of the study population´s ethnicity, which is known to significantly affect 25(OH)D levels and has been a bias in many previously published VitD studies (56). Therefore, our findings cannot be extrapolated to other ethnical groups without due caution. As this was an observational study, our findings would benefit from a larger sample size to increase the power and precision of the findings and consequently it will be reflected in narrowed confidence intervals.

To the best of our knowledge, this is the first study that determines the effect of total 25(OH)D, bioavailable 25(OH)D, free 25(OH)D, and % free 25(OH)D in individual follicles on embryo blastulation and ploidy status. In VitD deficient patients, the chance of having a euploid blastocyst increases as 25(OH)D-Serum/FF, bioavailable 25(OH)D-Serum/FF and free 25(OH)D-FF increase. In addition, VitD deficient patients unsurprisingly also derive benefit from a younger female age. Though VitD non-deficient patients have a significantly increased chance of obtaining euploid blastocysts, in contrast, individual increases in 25(OH)D-FF and bioavailable 25(OH)D-FF reduce the chance of obtaining euploid blastocysts. This study has clearly demonstrated that VitD plays a role in determining the ploidy status of blastocysts in a Middle Eastern population. Patients with adequate levels of VitD have a significantly increased chance of obtaining euploid blastocysts, while deficiency is associated with a lower likelihood to achieve a euploid blastocyst.

## Data Availability Statement

The raw data supporting the conclusions of this article will be made available by the authors, without undue reservation.

## Ethics Statement

The studies involving human participants were reviewed and approved by Ethics Committee of ART Fertility Clinics. Fertility Clinics, Abu Dhabi (UAE) (Research Ethics Committee REFA007). The patients/participants provided their written informed consent to participate in this study. Written informed consent was obtained from the individual(s) for the publication of any potentially identifiable images or data included in this article.

## Author Contributions

AAr and HF contributed to the conception and design of the study. AAr, IE, AB, and AP contributed to the data collection. AAr, ND, and AAb contributed to the data interpretation. AAr and ND drafted the manuscript. AAb, AB, BL, LM, CC, JG-V, and HF critically revised the manuscript for important intellectual content. All authors contributed to the article and approved the submitted version.

## Conflict of Interest

The authors declare that the research was conducted in the absence of any commercial or financial relationships that could be construed as a potential conflict of interest.
